# Effect of Genetically Low 25-Hydroxyvitamin D on Mortality Risk: Mendelian Randomization Analysis in 3 Large European Cohorts

**DOI:** 10.3390/nu11010074

**Published:** 2019-01-02

**Authors:** Thor Aspelund, Martin R. Grübler, Albert V. Smith, Elias F. Gudmundsson, Martin Keppel, Mary Frances Cotch, Tamara B. Harris, Rolf Jorde, Guri Grimnes, Ragnar Joakimsen, Henrik Schirmer, Tom Wilsgaard, Ellisiv B. Mathiesen, Inger Njølstad, Maja-Lisa Løchen, Winfried März, Marcus E. Kleber, Andreas Tomaschitz, Diana Grove-Laugesen, Lars Rejnmark, Karin M. A. Swart, Ingeborg A. Brouwer, Paul Lips, Natasja M. van Schoor, Christopher T. Sempos, Ramón A. Durazo-Arvizu, Zuzana Škrabáková, Kirsten G. Dowling, Kevin D. Cashman, Mairead Kiely, Stefan Pilz, Vilmundur Gudnason, Gudny Eiriksdottir

**Affiliations:** 1 Icelandic Heart Association, 201 Kopavogur, Iceland; thor@hi.is (T.A.); albertvs@umich.edu (A.V.S.); elias@hjarta.is (E.F.G.); v.gudnason@hjarta.is (V.G.); 2 Faculty of Medicine, School of Health Sciences, University of Iceland, 101 Reykjavik, Iceland; 3 Department of Internal Medicine, Division of Endocrinology and Diabetology, Medical University of Graz, 8036 Graz, Austria; martin.gruebler@gmx.net (M.R.G.); stefan.pilz@chello.at (S.P.); 4 Department of Cardiology, Medical University of Graz, 8036 Graz, Austria; andreas.tomaschitz@medunigraz.at; 5 Swiss Cardiovascular Center Bern, Department of Cardiology, Bern University Hospital, University of Bern, 3012 Bern, Switzerland; 6 Department of Laboratory Medicine, Paracelsus Medical University, 5020 Salzburg, Austria; keppel.martin@gmail.com; 7 Division of Epidemiology and Clinical Applications, National Eye Institute, Bethesda, MD 20892-1204, USA; mfc@nei.nih.gov; 8 Laboratory of Epidemiology and Population Sciences, National Institute on Aging, Bethesda, MD 20814, USA; tamara.b.harris@gmail.com; 9 Tromsø Endocrine Research Group, Department of Clinical Medicine, UiT The Arctic University of Norway, 9037 Tromsø, Norway; rolf.jorde@unn.no (R.J.); guri.grimnes@uit.no (G.G.); ragnar.joakimsen@uit.no (R.J.); 10 Tromsø Cardiovascular Research Group UNN, Department of Clinical Medicine, UiT The Arctic University of Norway, 9037 Tromsø, Norway; henrik.schirmer@uit.no; 11 Department of Community Medicine, UiT The Arctic University of Norway, 9037 Tromsø, Norway; tom.wilsgaard@uit.no (T.W.); inger.njolstad@uit.no (I.N.); maja-lisa.lochen@uit.no (M.-L.L.); 12 Brain and Circulation Research Group, Department of Clinical Medicine, UiT The Arctic University of Norway, 9037 Tromsø, Norway; ellisiv.mathiesen@uit.no; 13 Department of Neurology, University Hospital of North Norway, 9038 Tromsø, Norway; 14 Medical Clinic V, Mannheim Medical Faculty, University of Heidelberg, 68167 Mannheim, Germany; Winfried.Maerz@synlab.com (W.M.); Marcus.Kleber@medma.uni-heidelberg.de (M.E.K.);; 15 SYNLAB Academy, SYNLAB Holding Deutschland GmbH, P5, 7, D-68161 Mannheim or Gubener Straße 39, 86156 Augsburg, Germany; 16 Clinical Institute of Medical and Chemical Laboratory Diagnostics, Medical University of Graz, 8036 Graz, Austria; 17 NutriCard—Competence Cluster for Nutrition and Cardiovascular Health, Institute for Nutritional Science, Friedrich-Schiller-University, 07743 Jena, Germany; 18 Specialist Clinic for Rehabilitation Bad Aussee, 8990 Bad Aussee, Austria; 19 Department of Internal Medicine—Cardiology, Charité University Hospital Berlin, Campus Virchow Klinikum, 10117 Berlin, Germany; 20 Department of Endocrinology and Internal Medicine, Aarhus University Hospital, 8200 Aarhus, Denmark; jenngrov@rm.dk (D.G.-L.); lars.rejnmark@rm.dk (L.R.); 21 Amsterdam UMC, Vrije Universiteit Amsterdam, Department of Epidemiology and Biostatistics, Amsterdam Public Health, 1081 Amsterdam, The Netherlands; k.swart@vumc.nl (K.M.A.S.); nm.vanschoor@vumc.nl (N.M.v.S.); 22 Department of Health Sciences, Faculty of Sciences and Amsterdam Public Health Research Institute, Vrije Universiteit Amsterdam, 1081 Amsterdam, The Netherlands; ingeborg.brouwer@vu.nl; 23 Department of Internal Medicine, Endocrine Section, VU University Medical Center, 1081 Amsterdam, The Netherlands; P.Lips@vumc.nl; 24 Office of Dietary Supplements, National Institute of Health, Bethesda, MD 20892-7517, USA; semposch@gmail.com; 25 Department of Public Health Sciences, Loyola University Stritch School of Medicine, Maywood, IL 60153, USA; rdurazo@LUC.edu; 26 Cork Centre for Vitamin D and Nutrition Research, School of Food and Nutritional Sciences, University College Cork, Cork T12K8AF, Ireland; zuzkani@gmail.com (Z.Š.); 115224470@umail.ucc.ie (K.G.D.) K.Cashman@ucc.ie (K.D.C.); M.Kiely@ucc.ie (M.K.); 27 Department of Medicine, University College Cork, Cork T12K8AF, Ireland; 28 Irish Centre for Fetal and Neonatal Translational Research [INFANT], University College Cork, Cork T12K8AF, Ireland

**Keywords:** Vitamin D, standardized 25(OH)D, Mendelian randomization, mortality, cohorts, Individual Participant Data

## Abstract

The aim of this study was to determine if increased mortality associated with low levels of serum 25-hydroxyvitamin D (25(OH)D) reflects a causal relationship by using a Mendelian randomisation (MR) approach with genetic variants in the vitamin D synthesis pathway. Individual participant data from three European cohorts were harmonized with standardization of 25(OH)D according to the Vitamin D Standardization Program. Most relevant single nucleotide polymorphisms of the genes *CYP2R1* (rs12794714, rs10741657) and *DHCR7/NADSYN1* (rs12785878, rs11234027), were combined in two allelic scores. Cox proportional hazards regression models were used with the ratio estimator and the delta method for calculating the hazards ratio (HR) and standard error of genetically determined 25(OH)D effect on all-cause mortality. We included 10,501 participants (50.1% females, 67.1±10.1 years) of whom 4003 died during a median follow-up of 10.4 years. The observed adjusted HR for all-cause mortality per decrease in 25(OH)D by 20 nmol/L was 1.20 (95% CI: 1.15–1.25). The HR per 20 nmol/L decrease in genetically determined 25(OH)D was 1.32 (95% CI: 0.80–2.24) and 1.35 (95% CI of 0.81 to 2.37) based on the two scores. In conclusion, the results of this MR study in a combined sample from three European cohort studies provide further support for a causal relationship between vitamin D deficiency and increased all-cause mortality. However, as the current study, even with ~10,000 participants, was underpowered for the study of the effect of the allele score on mortality, larger studies on genetics and mortality are needed to improve the precision.

## 1. Introduction

Vitamin D is critical for bone and mineral metabolism. Vitamin D status including deficiency is assessed by the concentration of serum 25-hydroxyvitamin D (25(OH)D) and vitamin D deficiency, as measured by a low 25(OH)D concentration, as well as calcium deficiency are causally related to the development of skeletal disease: rickets in children and osteomalacia in adults (IOM/SACN/EFSA). In addition, vitamin D receptors (VDRs) have also been identified in almost all extra-skeletal tissues suggesting a widespread role of vitamin D for human health. Moreover, 25(OH)D has been associated with common chronic diseases. But as the majority of clinical trials failed to show a health benefit of vitamin D supplementation in lowering the risk of chronic diseases other than rickets and osteomalacia the question is raised whether vitamin D deficiency is actually a causal factor for those other chronic diseases [1,2]. 

Observational studies have, by the majority, shown an association of low serum 25(OH)D with an increased risk of all-cause mortality [3,4,5,6,7,8,9]. In line with this, meta-analyses of randomized controlled trials (RCTs) have reported that vitamin D_3_ supplementation in particular together with calcium reduces mortality with small, yet significant, effects [10,11,12,13]. Due to small effect sizes and several other limitations such as incomplete follow-up data it still remains unclear whether higher circulating levels of serum 25(OH)D have a causal effect on improving survival. Considering the high prevalence of vitamin D deficiency [14] and the relatively simple way of improving vitamin D status by, for example, food fortification or supplementation it is an important public health issue to identify the role of vitamin D in the prevention of premature mortality due to all-causes. 

Genetic studies can help in the prediction of the causal effects of 25(OH)D on clinical endpoints through Mendelian randomization (MR) analyses using the method of instrumental variables (IV). This is done to address the problem of confounding and reverse causation in epidemiology [15]. Shortly explained, MR studies are based on the principle that the presence or absence of certain genetic single nucleotide polymorphisms (SNPs) of a particular gene is distributed randomly in the community and therefore its association with the outcome of interest allows, implicitly, assumptions on causality. MR studies are useful to address the question of causality, and reverse causation, because an association between genetically determined 25(OH)D levels and mortality is very likely to be causal as the genetic variation in 25(OH)D levels is not confounded by lifestyle factors. In an MR study including 95,766 participants from Copenhagen, Denmark, Afzal et al. [16] found a causal effect of 25(OH)D on all-cause mortality using an allele score, a finding that needs confirmation in other independent cohorts. This was reiterated in an editorial in the same issue, where Welsh and Sattar [17] encouraged additional MR studies.

The aim of the present study, which is part of the Food-Based Solutions for Optimal Vitamin D Nutrition and Health through the Life Cycle (ODIN) project (www.odin-vitd.eu), was to perform a MR study on 25(OH)D and mortality in three European cohorts. The novelty of our investigation is the standardization of the 25(OH)D values and harmonization of the phenotypes from the cohorts to allow accurate analyses of the individual participant data by serology and genotype. 

## 2. Materials and Methods

### 2.1. Cohorts and Data Collection

Individual participant data from three European cohorts, AGES (Iceland), LURIC (Germany) and Tromsø (Norway), were combined. All cohorts were of European ancestry. The three cohort studies included in the present analyses were selected within the ODIN consortium based on the availability of genetic data, and follow-up data for mortality analyses. These studies are the Age, Gene/Environment Susceptibility (AGES) Reykjavik Study [18], the Ludwigshafen RIsk and Cardiovascular Health (LURIC) Study [19] and the Tromsø Study [20]. The phenotypes from the cohorts were carefully harmonized for analysis [8] and all had analyzed SNPs in genes coding for key metabolic processes in the synthesis pathway of vitamin D [21]. We did not include SNPs of genes in the vitamin D transport and metabolism pathway as Dastani et al. [22] have shown that the biological role of vitamin D is independent of circulating levels of vitamin D binding protein and that these enzymes may have pleiotropic- or unclear effects on biologically active 25(OH)D levels [23]. Each cohort is described in detail in Supplement 1 as well as in previous publications of the ODIN project [8,14]. Serum 25(OH)D was analyzed at each site (using CLIA, RIA, and ECLIA, methods in AGES, LURIC, Tromsø respectively) and the measurements were standardized by means of the United States‘ National Institutes of Health led International Vitamin D Standardization Program (VDSP) [24].

In brief, representative samples were re-analyzed at University College, Cork, Ireland using a CDC-certified liquid chromatography–tandem mass spectrometry method, master regression equations of the old versus re-analyzed serum 25(OH)D established, and these tailored algorithms applied to each cohort’s data to standardize the 25(OH)D values [14]. Phenotypes were also harmonized across cohorts [8]. The descriptions of the genotyping methods used in each cohort are shown in Supplement 2. SNPs in/near genes connected to the synthesis pathway of 25(OH)D [25,26] were provided by the three cohorts. For the statistical analysis four SNPs from loci near genes involved in the vitamin D synthesis pathway (i.e., genes for dermal vitamin D synthesis and 25-hydroxylation of vitamin D) were combined to create two genetic scores. The first score (G1) is the count of the 25(OH)D alleles for the SNPs, rs12794714 and rs12785878. They were chosen because of availability in the three cohorts and they gave the strongest genetic association with 25(OH)D levels in our data. The second score (G2) was generated in a similar way to the genetic score in the study from Copenhagen [16]. The count of the alleles of variants in DHCR7 (rs12785878, rs11234027), and variants in CYP2R1 (rs12794714, rs10741657) were added together. 

### 2.2. Statistical Methods

General linear models were used to estimate the association between the genetic scores, denoted by G, and the exposure 25(OH)D, denoted by X. The regression coefficient for the difference in X per allele difference in G was denoted by ${\hat{\beta }}_{GX}$. 

In the observational analysis a Poisson regression model was used to estimate the absolute mortality rate by level of 25(OH)D. The functional shapes used were linear, log-linear and a spline function. The Cox proportional hazards regression model was used to estimate the observational hazard ratio for mortality (the outcome Y) for per 20 unit difference in 25(OH)D.

The Cox proportional hazards regression model was used to estimate the hazard ratio for mortality (the outcome Y) for per allele change in the genetic score G. The log-hazard ratio estimate was denoted by ${\hat{\beta }}_{GY}$.

In the causal Mendelian randomization analysis an IV approach was used with the genetic score (G) as an instrumental variable and a determinant of 25(OH)D as the exposure (X). The estimate of the genetically determined hazard ratio (HR) of mortality, for a decrease in 25(OH)D, was based on the Wald ratio estimator with confidence interval computed by the delta method, also known as Fieller’s method for a ratio [27,28]. In other words, the causal estimate of the HR for death for *k* unit change in 25(OH)D was $exp(k{\hat{\beta }}_{GY}/{\hat{\beta }}_{GX}$), where exp() is the exponential function. The standard error of the ratio estimator from the delta method is a function of ${\hat{\beta }}_{GY}$_,_ ${\hat{\beta }}_{GX}$_,_ and the standard errors $SE\left({\hat{\beta }}_{GY}\right)$ and $SE\left({\hat{\beta }}_{GX}\right)$.

(1) $$Standard\;error≅\sqrt{\frac{SE{\left({\hat{\beta }}_{GY}\right)}^{2}}{{\hat{\beta }}_{GX}^{2}}+\frac{{\hat{\beta }}_{GY}^{2}SE{\left({\hat{\beta }}_{GX}\right)}^{2}}{{\hat{\beta }}_{GX}^{4}}}$$

The IV analysis was performed both assuming linearity, and by dividing 25(OH)D levels into six categories, <30 nmol/L, 30–39.9 nmol/L, 40–49.9 nmol/L, 50–74.9 nmol/L, 75–99.9 (reference category), and 100–150 nmol/L [29]. We limited the range of 25(OH)D under study to 0–150 nmol/L. This resulted in omitting data on 6 individuals with 2 deaths. It was impossible to study the effect of 25(OH)D for values greater than 150 nmo/l with only 6 individuals and 2 deaths representing that range. We adjusted for the potential measured confounding variables: study (categorical variable), age, sex, season of blood draw (categorical), body mass index (BMI), active smoking status, diabetes mellitus, arterial hypertension, history of cardiovascular disease (CVD), and history of cancer. 

We tested if the genetic scores G1 and G2 were associated with any of the 10 measured confounding variables in the 3 cohorts at the significance level 0.05/(3 × 10) = 0.0017. We also tested if associations between G1, G2, and X; X and Y; and G1, G2, and Y were the same for men and women. In other words, we tested for effect modification by sex, using interaction terms with sex and the predictor variable in each case. 

## 3. Results

Basic descriptive information of the study participants from each cohort is shown in Table 1. In general, the cohorts are similar with regard to baseline characteristics. The LURIC cohort differs from the other cohorts in having fewer females, more CVD and diabetes, and a lower average serum 25(OH)D concentration. There are more smokers in the Tromsø cohort than in the other cohorts. LURIC and Tromsø had a wider age range than AGES.

The percentage of participants with level of serum 25(OH)D <30 nmol/L was 12.1% and 24% had level <40 nmol/L.

Associations of the genetic scores with any of the potential confounding variables were not statistically significant. Associations of each SNP and the genetic score, adjusted for study, with serum measured 25(OH)D levels were highly significant as shown in Table 2. The multiple linear regression model estimate of the effect size of the association of the genetic score (G1) and 25(OH)D was −1.33 nmol/L (95% CI −1.66 to −1.00) for each allele. For G2 the estimate was −0.71 nmol/L (95% CI −0.90 to −0.52). This estimate was denoted by ${\hat{\beta }}_{GX}$ for each score. The F-statistic for G1 was 52 and 0.49 % of the variation in serum 25(OH)D was explained, and for G2 the F-statistic was 47.4 with 0.46% of the variation explained. 

**Figure 1:**
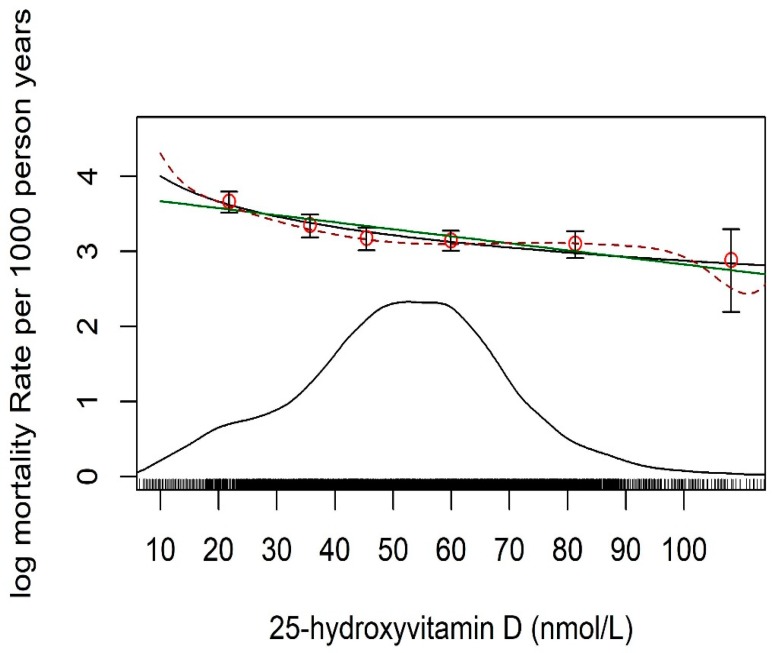
Mortality rate (log-scale) for 10 years from all causes by level of standardized 25-hydroxyvitamin D. A rug and density plot are superimposed to demonstrate where most of the 25(OH)D measurements lie. Fitted associations are shown as linear (green line), log-linear (black line), and spline (broken line).

Due to the small sample size of the three cohorts we do not show individual cohort results and restrict our mortality analysis to the combined individual participant data of the entire sample. The genetic association with levels of 25(OH)D and the association between levels of 25(OH)D and mortality had consistent direction in all 3 cohorts. The median follow-up time was 10.4 years (interquartile range: 8.6 to 17.1 years) and 4003 participants died during this time for a total of 117,038 person years. A negative association of serum 25(OH)D levels with all-cause mortality rate (on log-scale) is shown in Figure 1; and it was found to be log-linear rather than linear (*p* < 0.001). 

However, the deviation from linearity was not severe, and only noticeable for low values (<15 nmol/L). For comparison to other published studies, the results from the linear association and by using categories for 25(OH)D were presented. The log-hazard ratio per one allele score increase (G1) for all-cause mortality was ${\hat{\beta }}_{GY}$= 0.0183 (95% confidence interval (CI): −0.0146 to 0.0511) with the HR = 1.0184 (95% CI: 0.9855 to 1.0524). For G2 it was ${\hat{\beta }}_{GY}$= 0.01074 (95% confidence interval (CI): −0.0076 to 0.0291) with the HR = 1.0108 (95% CI: 0.9924 to 1.0295).

The observational estimate of the HR of mortality for a decrease in 20 nmol/L 25(OH)D was 1.20 (95% CI: 1.15 to 1.25). Based on the G1 score the genetically determined HR for a decrease in 20 nmol/L 25(OH)D, was exp(20 × *β_GY_*/*β_GX_*) = 1.32 with a 95% CI of 0.80 to 2.24. Based on G2 score it was 1.35 with 95% CI of 0.81 to 2.37.

HR for mortality by categories of 25(OH)D is shown in Table 3. The observational HR for mortality was highest with 1.76 (95% CI: 1.50 to 2.07) in individuals with 25(OH)D <30 nmol/L compared to individuals with 75 to 99.9 nmol/L. The HR of genetically determined 25(OH)D was higher compared to the HR for serum 25(OH)D, but did not reach statistical significance. Results are shown in Table 3.

We did not find sex to be an effect modifier for any of these associations.

## 4. Discussion

In this MR study involving individual participant data from three European cohorts we observed an association of low 25(OH)D and increased mortality with similar risk estimates for serum 25(OH)D and for genetically determined 25(OH)D levels.

Our results are consistent with the only other large MR study on vitamin D and mortality, performed by Afzal et al. [16] derived from three Danish cohorts. Our observational estimate of the HR for mortality per decrease in 20 nmol/L 25(OH)D levels was 1.20 (95% CI: 1.15 to 1.25). Theirs was 1.19 (95% CI: 1.14−1.25). Our HR for mortality per 20 nmol/L decrease in genetically determined 25(OH)D levels was 1.32 (95% CI: 0.84 to 2.20) and 1.35 with 95%CI of 0.81 to 2.37. Theirs was 1.30 (95% CI: 1.05−1.61). This confirms that both studies have similar point-estimates and effect sizes.

The limiting factor in our study is the size of the cohort to estimate the IV and mortality association, not the size of the gene and exposure association. This has been demonstrated in a general setting by Pierce and Burgess [30]. The way forward would either be to increase the size of the cohort with IV and mortality information and treat our cohort as a subsample, or get an estimate from independent sample and approach the analysis as 2-sample estimator. 

These consistent findings along with the fact that in both studies risk estimates were similar for measured as well as genetically determined 25(OH)D levels suggest that the association between vitamin D deficiency and mortality is causal. Nevertheless, the effect of the genetic score on the 25(OH)D variation was low in our study, but the F statistic result suggests that it has potential to be a good instrument to detect causal relationships. Afzal et al. [16] analyzed SNPs in the same two genes as in our genetic score and reported that approximately 1% of the 25(OH)D variation was explained by their genetic score. This was also similar to the variation explained in our study. We have to acknowledge that our cohort sample size per se is underpowered for statistically significant results. The similarity of the findings and effect sizes of these two MR studies, despite some obvious differences in the cohort characteristics such as smoking prevalence and CVD burden, lends strong support in favor of a causal vitamin D mortality relationship. 

Large genome wide association studies (GWAS) have found a number of SNPs significantly associated with 25(OH)D near genes in the vitamin D metabolic pathway [21,26,31]. The SNPs studied in our present work are in the vitamin D synthesis pathway. These SNPs have been chosen in many of the MR studies involving vitamin D, and allelic scores have been found to be more powerful than individual SNPs as an instrument in such studies [25]. Many conditions in addition to mortality have been studied in MR analyses attempting to determine causal relationships of vitamin D with bone mineral density [32], type 2 diabetes [33], age-related macular degeneration [34], hypertension [35], ischaemic heart disease [36], obesity [37,38], brain function [39], and seven types of cancer [40,41]. Mainly negative results were obtained [32,33,34,36,38,40,41] or further research is needed to confirm positive results [35,37,38]. A report from the SUNSHINE consortium [42] enrolling 33,996 patients implicated 25(OH)D as a causal risk factor for Alzheimer’s disease, whereas in the same population genetically lowered 25(OH)D was not associated with increased risk of CAD. The SNPs described 2.44% of the variance of 25(OH)D, higher than in our study.

The results presented here support the argument posited by Schöttker et al. [43] suggesting that vitamin D supplementation through food or vitamin supplements might help people to be more resilient to premature mortality, though the mechanism remains unclear. Since the present study is underpowered to study specific causes of death, we can only speculate about the underlying pathophysiological mechanisms and the life threatening diseases that were the main drivers for our results. Currently there is inconsistent evidence that vitamin D supplementation has any clinically meaningful effect on decreasing falls, cancer and CV deaths in community dwelling adults. But concomitant chronic diseases such as CVD or neurological diseases may also play a role in the body‘s need for vitamin D to maintain homeostasis. Before advocating in support of vitamin D supplementation or any determination of the ideal vitamin D level, it will be important to wait for further results from on-going, population-based vitamin D randomized clinical trials (RCTs) [44], although a major limitation of many vitamin D RCTs is the inclusion of participants regardless of 25(OH)D levels. Our results show a significant observed mortality increase at levels below 40 nmol/L 25(OH)D. Recently published vitamin D RCTs did not report health benefits of vitamin D but they might have been underpowered to detect significant overall effects, or clinically meaningful subgroup differences, due to their sample size and a high prevalence of participants with sufficient 25(OH)D levels [45,46,47,48]. For example, in the VITamin D and OmegA-3 trial (VITAL), 25,871 participants with a mean baseline serum 25(OH)D concentration of 77 ± 25 nmol/L were randomized to 2000 international units vitamin D or placebo. During a median follow-up of 5.3 years there was no effect on total mortality (HR: 0.99; 95% CI: 0.87 to 1.12) [48]. Furthermore, while RCTs usually have a limited study duration, MR studies indicate effects of lifelong exposure.

## 5. Strengths and Limitations

The strength of this study lies in the standardization of the 25(OH)D measurement, the inclusion of cohorts from three different countries and harmonization of phenotypes across the three cohorts. Standardization of 25(OH)D values reduces interference by methodological problems of 25(OH)D measurements that can negatively impact on the ability to compare and contrast findings across studies. Using individual participant data with harmonized phenotypes is also an advantage for combining the data of the three cohorts into a single, perhaps more generalizable, sample. 

A limitation of the current study is that there is only one measurement of 25(OH)D, collected at a single time-point. The current study was underpowered for the study of the effect of the allele score on mortality. The effect per allele was small and the current study would have needed five to six times larger cohort (or 50,000 to 60,000 persons) to be able to estimate the IV mortality association with enough precision for the Mendelian randomization analysis to be realistically able to reach statistical significance. The cohorts included only people of European ancestry and therefore the results may not apply to other ethnic groups. 

## 6. Conclusions

In conclusion, the results of this MR study may argue in favor of a causal relationship between vitamin D deficiency and increased all-cause mortality in a combined sample from three European cohort studies. These data support a previous Danish MR study with similar effects size. It is also in line with the dose-response seen in observational epidemiological studies as well as meta-analyses of vitamin D RCTs, which suggest that low 25(OH)D levels may be detrimental for survival. These findings on vitamin D and mortality deserve consideration in the public health discussion regarding the value, design, and implementation of innovative approaches to improve the vitamin D status of the general population. 

## Figures and Tables

**Table 1:** General characteristics of the three individual cohorts and the combined sample.

Characteristic	Total Sample for Analysis		AGES		LURIC		Tromsø	
	*N* = 10501		*N* = 3172		*N* = 2864		*N* = 4465	
	N/Mean	%/SD	N/Mean	%/SD	N/Mean	%/SD	N/Mean	%/SD
Sex (females) *	5266	50.1	1834	57.8	866	30.2	2566	57.5
Age	67.1	10.1	76.4	5.5	62.9	10.5	63.2	7.7
BMI, kg/m^2^	26.8	4.2	27.1	4.4	27.4	4.0	26.2	4.2
Season blood drawn ^a^								
spring	3011	28.7	810	25.5	602	21.0	1599	35.8
summer	1328	12.7	448	14.1	711	24.8	169	3.8
fall	3180	30.3	1086	34.2	880	30.7	1214	27.2
winter	2982	28.4	828	26.1	671	23.4	1483	33.2
25(OH)D, nmol/L^b^	51.7	18.1	57.1	17.8	42.3	22.7	54.0	11.4
LDL, mmol/L	3.8	1.2	3.5	1.0	3.0	0.9	4.5	1.2
Glucose, mmol/L^c^	5.7	1.5	5.8	1.1	5.6	1.8	-	-
SBP, mmHg	144.3	22.5	142.5	20.3	141.3	23.6	147.4	22.9
Arterial hypertension *^,d^	8101	77.2	2558	80.7	2677	93.5	2866	65.2
Active smoker *	2418	23.0	403	12.7	540	18.9	1475	33
Diabetes *^,e^	1482	14.3	365	11.5	943	32.9	174	4.0
Previous cancer *	1034	9.9	489	15.4	209	7.3	336	7.5
CVD history ^*,f^	2400	22.9	613	19.3	1349	47.1	438	9.8
Death *	4003	38.1	1184	37.3	855	29.9	1964	44.0

AGES: Age, Gene/Environment Susceptibility (AGES) Reykjavik Study; LURIC: Ludwigshafen RIsk and Cardiovascular Health (LURIC) Study; Tromsø: Tromsø Study; BMI = body mass index; LDL = low density lipoprotein, SBP = systolic blood pressure; CVD = Cardiovascular disease. * Values are number and percent for categorical variables. ^a^ Season of baseline blood sampling was defined as spring (March to May), summer (June to August), autumn (September to November), and winter (December to February). ^b^ Standardized 25(OH)D values for all three cohorts by means of the Vitamin D Standardization Program (VDSP), excluding participants >150 nmol/L (*n* = 6). ^c^ Fasting glucose. ^d^ Arterial hypertension at baseline was defined as: Participants already on antihypertensive drug treatment, physician-reported, self-reported HTN, office systolic and/or diastolic blood pressure of equal to or higher than 140 and/or 90 mmHg (ICD-9: 401,405; ICD-10: I10,I15). ^e^ Diabetes mellitus at baseline was defined as those participants on glucose lowering drugs, physician-reported, self-reported or according to ADA: fasting glucose ≥ 7.0 mmol/L, 2 h postload glucose ≥ 11.1 mmol/L or HbA1c ≥ 6.5% (ICD-9: 250; ICD-10: E10-E14). ^f^ History of CVD at baseline was defined as positive history of myocardial infarction and/or stroke.

**Table 2:** SNP and genetic score association, adjusted only for study, with standardized 25-hydroxyvitamin D(25(OH)D). Beta represents the effect per allele count. SNP: single nucleotide polymorphism.

GENE	Activity	SNP	Beta	*p*-Value
*DHCR7/NADSYN1*	Regulates vitamin D precursor	rs12785878	−1.18 nmol/L	1.77 × 10^−6^
*CYP2R1*	25-hydroxylation of vitamin D	rs12794714	−1.26 nmol/L	1.34 × 10^−7^
Genetic score (G1)		SUM	−1.25 nmol/L	5.99 × 10^−13^
*DHCR7/NADSYN1*	Regulates vitamin D precursor	rs12785878 rs11234027	−0.63	8.68 × 10^−6^
*CYP2R1*	25-hydroxylation of vitamin D	rs12794714 rs10741657	−0.70	5.07 × 10^−8^
*Genetic score (G2)*		SUM	−0.66	6.19 × 10^−12^

**Table 3:** HR of mortality by categories of 25-hydroxyvitamin D (25(OH)D, nmol/L), and estimates by categories assuming linearity for observational and MR estimates.

Model		<30	30−39.9	40−49.9	50−74.9	75−99.9 (Ref)	100−150
Basic information	Median nmol/L	21.8	35.7	45.4	59.9	81.3	108.1
	*N*	1303	1230	2174	4882	824	88
	Deaths, *n*	574	485	869	1808	250	17
	Person years	10592	12452	26904	57820	8434	837
	Death Rate *	54.2	38.9	32.3	31.3	29.6	20.3
Observational estimate by categories	HR	1.76	1.29	1.11	1.06	1.00	0.83
	95% CI	1.50−2.07	1.11−1.51	0.95−1.28	0.93−1.22		0.51−1.36
Observational estimate by linearity at midpoint	HR	1.71	1.51	1.38	1.21	1.00	0.79
	95% CI	1.51−1.94	1.37−1.66	1.28−1.49	1.16−1.27		0.74−0.83
MR (G1) estimate by linearity at midpoint	HR	2.26	1.87	1.64	1.34	1.00	0.69
	95% CI	0.52−10.96	0.60-6.26	0.67−4.24	0.79−2.36		0.34−1.55
MR (G2) estimate by linearity at midpoint	HR	2.45	1.99	1.72	1.38	1.00	0.67
	95% CI	0.52−12.98	0.61−7.13	0.68−4.70	0.79−2.51		0.32−1.34

Analysis was done using Cox proportional hazards ratio adjusting for age, sex, study, season of blood draw, BMI, smoking, diabetes mellitus, arterial hypertension, history of cardiovascular disease, and history of cancer. HR = Hazards ratio; MR = Mendelian randomization; Ref = Reference group. * Per 1000 person years.

## Supplementary Materials

The following are available online at http://www.mdpi.com/2072-6643/11/1/74/s1, Supplement 1: Study details, Supplement 2: Genotyping methods, Supplement 3: Additional funding sources.
