# Supplementary material for: Effect of Genetically Low 25-Hydroxyvitamin D on Mortality Risk: Mendelian Randomization Analysis in 3 Large European Cohorts
Source: Nutrients. 2019 Jan 2;11(1):74. doi: 10.3390/nu11010074 (PMC6356674; doi:10.3390/nu11010074)
Supplement: Supplementary file 1 [file nutrients-11-00074-s001.zip › nutrients-402488-SI.pdf]

Supplement 1 Study details

1.Age, Gene/Environment Susceptibility (AGES) Reykjavik Study

The AGES Reykjavik Study is conducted by a collaboration of the the Icelandic Heart Association and National Institute on Aging, National Institutes of Health, Bethesda, Maryland, USA . The AGES-Reykjavik sample is drawn from an established population-based cohort, the Reykjavik Study, and presents a longitudinal population-based study conducted in Iceland, situated at latitude of about 64°N. AGES-Reykjavik examinations began in 2002. At that time, there were 11549 previously examined Reykjavik Study cohort members still alive. At the end of AGES-Reykjavik examinations in February 2006, the study compromised 5764 survivors of the Reykjavik Study cohort. The AGES-Reykjavik examination is a single wave examination, completed in three clinic visits, with a participant's full examination completed within a four to six week time window (1). Of the 5764 individuals, there were missing values for 25(OH)D (n=245), BMI (n=8), mortality follow-up (n=1), and genotypes so that the final sample compromised 5406 individuals.

2. Ludwigshafen RIsk and Cardiovascular Health (LURIC) Study

The LURIC Study is a prospective, hospital-based cohort study among 3316 study participants who were routinely referred to a tertiary care medical centre in south-west Germany, situated at 49°N, between 1997 and 2000 (2). Inclusion criteria were European ancestry and with no other acute illness except for the availability of a coronary angiogram (ACS). Exclusion criteria were any acute illness other than ACS, any chronic disease where non-cardiac disease predominated and a history of malignancy within the past five years. Participants were continuously followed up with respect to fatal events. As there were missing values for 25(OH)D (n=17) and genotypes (n = 29), the final sample compromised 3270 individuals.

3. Tromsø Study

The Tromsø Study, conducted by the University of Tromsø in cooperation with the National Health Screening Service, is a longitudinal population-based study in results of the municipality of Tromsø, Norway, situated at 69°N (3). The baseline visit for the present analysis is the fourth survey, which was performed in 1994–1995, with repeated follow-up surveys conducted at six to seven years intervals. Of the 26956 participants, there were missing values for 25-hydroxyvitamin D (25[OH]D) (n=19796), and body mass index (BMI) (n=15), only a subset was genotyped so the final sample for the present study compromised 4533 individuals.

Supplement 2 Genotyping methods

1. Age, Gene/Environment Susceptibility (AGES) Reykjavik Study

DNA was isolated from peripheral blood lymphocytes according to the method developed by Scotlab Bioscience (Kirshaws Road, Coatbridge, Strathclyde, UK). GWAS was performed at the Neurogenetics Lab, National Institute on Aging, NIH, Bethesda, MD, USA on an Illumina 370HuCNV platform. The Illumina Beadstudio was used for calling and the call rate was <95%, pHWE<10-6. Mach version 1.0.16 was used for imputation from HapMap release 22 (build 36) (4).

Study samples were also processed on the HumanExome BeadChip v1.0 (Illumina, Inc., San Diego, CA) querying 247,870 variable sites. The Illumina GenomeStudio v2011.1 software was utilized with the GenTrain 2.0 clustering algorithm for calling. Genomic DNA study samples and HapMap controls with call rates .99% (n= 55,142) were used to define genotype clusters. DBNSFP v2.0 was used to annotate the variants (5).

2.Ludwigshafen RIsk and Cardiovascular Health (LURIC) Study

Genotyping was performed by the LURIC study group at the Synlab Clinical Diagnostics Laboratory in Heidelberg and at the Mannheim Institute of Public Health in Mannheim using the Affymetrix 6.0 array. Genotypes were called using the Birdseed v2 algorithm. Samples with a call rate < 95% and SNPs with a call rate <98%, a minor allele frequency < 1% or pHWE < 10-4 were filtered out before imputation. Imputation was performed using MACH with the HapMap release 22 (build 36) reference panel.

3. Tromsø Study

All genotyping was performed by KBioscience (http://www.kbioscience.co.uk) using KASP (KBioScience Allele-Specific Polymorphism) SNP genotyping system. KASP is a

competitive allele-specific PCR incorporating a FRET (Fluorescence Resonance Energy Transfer) quencher cassette (3).

Supplement 3 Additional funding sources

Age, Gene/Environment Susceptibility Reykjavik Study: This study has been funded by NIH contract N01-AG012100, the NIA Intramural Research Program, Hjartavernd (the Icelandic Heart Association), and the Althingi (the Icelandic Parliament), an Intramural Research Program Award (ZIAEY000401) from the National Eye Institute, an award from the National Institute on Deafness and Other Communication Disorders (NIDCD) Division of Scientific Programs (IAA Y2-DC_1004-02), The study is approved by the Icelandic National Bioethics Committee, VSN: 00-063.

Ludwigshafen RIsk and Cardiovascular Health Study: None.

Tromsø Study: None.

The funders had no role in study design, data collection and analysis, decision to publish, or preparation of the manuscript.

Conflict of interest. Paul Lips reports a competing interest: Advice to Friesland Campina, dairy industry. All other authors have nothing to declare.

Disclaimer

The findings and conclusions in this manuscript are those of the authors and do not necessarily represent the official views or positions of the U.S. National Institutes of Health or the Department of Health and Human Services.

REFERENCES

1. Harris TB, Launer LJ, Eiriksdottir G, Kjartansson O, Jonsson PV, Sigurdsson G, et al. Age, Gene/Environment Susceptibility-Reykjavik Study: multidisciplinary applied phenomics. American journal of epidemiology. 2007;165(9):1076-87. Epub 2007/03/14.

2. Winkelmann BR, Marz W, Boehm BO, Zotz R, Hager J, Hellstern P, et al. Rationale and design of the LURIC study--a resource for functional genomics, pharmacogenomics and long-term prognosis of cardiovascular disease. Pharmacogenomics. 2001;2(1 Suppl 1):S1-73. Epub 2001/03/22.

3. Jorde R, Schirmer H, Wilsgaard T, Joakimsen RM, Mathiesen EB, Njolstad I, et al. Polymorphisms related to the serum 25-hydroxyvitamin D level and risk of myocardial infarction, diabetes, cancer and mortality. The Tromso Study. PloS one. 2012;7(5):e37295. Epub 2012/06/01.

4. Psaty BM, O'Donnell CJ, Gudnason V, Lunetta KL, Folsom AR, Rotter JI, et al. Cohorts for Heart and Aging Research in Genomic Epidemiology (CHARGE) Consortium: Design of prospective meta-analyses of genome-wide association studies from 5 cohorts. Circulation Cardiovascular genetics. 2009;2(1):73-80. Epub 2009/12/25.

5. Grove ML, Yu B, Cochran BJ, Haritunians T, Bis JC, Taylor KD, et al. Best practices and joint calling of the HumanExome BeadChip: the CHARGE Consortium. PloS one. 2013;8(7):e68095. Epub 2013/07/23.
